# The Three Laws of Neurorobotics: A Review on What Neurorehabilitation Robots Should Do for Patients and Clinicians

**DOI:** 10.1007/s40846-016-0115-2

**Published:** 2016-02-09

**Authors:** Marco Iosa, Giovanni Morone, Andrea Cherubini, Stefano Paolucci

**Affiliations:** Clinical Laboratory of Experimental Neurorehabilitation, IRCCS Fondazione Santa Lucia, Via Ardeatina 306, 00179 Rome, Italy; CNRS-UM LIRMM UMR 5506, IDH group, 161 Rue Ada, 34392 Montpellier, France

**Keywords:** Rehabilitation, Robotic training, Neuroscience, Ethics, Medical robots

## Abstract

Most studies and reviews on robots for neurorehabilitation focus on their effectiveness. These studies often report inconsistent results. This and many other reasons limit the credit given to these robots by therapists and patients. Further, neurorehabilitation is often still based on therapists’ expertise, with competition among different schools of thought, generating substantial uncertainty about what exactly a neurorehabilitation robot should do. Little attention has been given to ethics. This review adopts a new approach, inspired by Asimov’s three laws of robotics and based on the most recent studies in neurorobotics, for proposing new guidelines for designing and using robots for neurorehabilitation. We propose three laws of neurorobotics based on the ethical need for safe and effective robots, the redefinition of their role as therapist helpers, and the need for clear and transparent human–machine interfaces. These laws may allow engineers and clinicians to work closely together on a new generation of neurorobots.

## Introduction

### Controversial Effectiveness of Robots for Neurorehabilitation

The first robots used for neurorehabilitation were developed in the 1980s [1, 2], their potential was claimed in the 1990s [3–5], and robotic exoskeletons started to spread in the 2000s [6, 7]. However, their is still debate on the effectiveness of robots in neurorehabilitation.

Contrasting results were obtained in different studies about neurorehabilitation robot efficacy [8–11], even though the results of some randomized controlled trials performed on wide samples showed significant improvements in the outcome of robot-assisted therapy with respect to usual care [12, 13]. Meta-analyses have only partially helped in clarifying the objective effectiveness of robotic training, with most results being inconclusive. A 2008 Cochrane review on post-stroke arm training robots [14] concluded its analysis on 11 studies (328 subjects) by stating that: “patients who receive electromechanical and robot-assisted arm training after stroke are not more likely to improve their activities of daily living, but arm motor function and strength of the paretic arm may improve”. The same authors further updated their Cochrane review in 2012 [15], including 19 trials (666 subjects), concluding: “Patients who receive electromechanical and robot-assisted arm training after stroke are more likely to improve their generic activities of daily living. Paretic arm function may also improve, but not arm muscle strength”. These results were hence in opposition with those obtained previously. Although the second Cochrane review should be considered more reliable, given the higher number of trials and enrolled subjects, the contrasting results (also in terms of muscle strength) lead to confusion.

Cochrane reviews on walking rehabilitation performed using robots also provide inconsistent results. A Cochrane review, as well as its update [16, 17], reported higher probability of recovery in patients who receive electromechanical-assisted gait training in combination with physiotherapy, whereas another Cochrane review [18] reported similar recovery probabilities for patients with and without treadmill training (i.e., with and without body weight support).

Besides effectiveness, three other aspects deserve attention. Firstly, these Cochrane reviews analysed electromechanical devices and robots as a single and homogeneous field. In fact, electromechanical devices developed for neurorehabilitation (e.g., treadmill with body weight support or Gait Trainer (Reha-Stim, Berlin, Germany)) are often but improperly considered members of the robot family [19]. This is a major concern for the designers of robot-therapy systems, who have failed so far to provide a comprehensive and agreed-on framework for the correct classification of these devices [20]. A second aspect deserving attention is that many studies about the efficacy of specific devices were published after their commercialization. This approach is inconceivable in other medical fields, for example pharmacology. The third point to take into account is that effectiveness should be referred not only to the device per se, but also to the specific patient groups targeted by the therapy [21–23], and to the timing and protocol adopted for that device [24]. This point was highlighted by Mehrholz et al. [16]: the correct use of new technologies must rely on the information regarding the types of patients and the phase of rehabilitation that will benefit from specific technologies. For example, patients with more severe impairments in the motor leg can benefit more from robotic-assisted therapy, in combination with conventional therapy, than from conventional therapy alone. This likely occurs because, in the case of very impaired patients, robotic devices, increase the therapy intensity with respect to conventional ones [21, 22]. Conversely, patients with greater voluntary motor function in the affected limb can perform intensive training also in conventional therapy. For these patients, neurorehabilitators may prefer less constrained, more ecological, and more variable exercises [25]. Physical condition is not the only factor determining the best class of neurorobot users: the patient psychological profile can also be important in attaining superior motor outcomes with robot training compared to conventional therapy [24].

These results have led to a proposal of a change in the research question about the effectiveness of robot devices: “instead of asking ourselves whether robotic devices are effective in rehabilitation, we should determine who will benefit more from robotic rehabilitation” [25]. Inclusion and exclusion criteria are not the only characteristics to be determined in the design of a rehabilitation protocol when a robot is used. Few studies have focused on the definition of guidelines for an effective selection of movement parameter values (such as joint angles, speeds, applied forces, and torques) and for better timing of robot therapy administration, both tailored on the patient’s capacities and needs.

However, before further discussing the issue of effectiveness, and the reasons of the limited credit that is given to neurorobots, it is fundamental to clarify the difference between robots and electromechanical devices by defining what a neurorobot is.

### What is a Neurorobot?

Some cooking machines are commonly called robots by manufacturers and end-users. However, no one calls a mixer a robot. This does not depend on machine complexity: a car is usually more sophisticated than a cooking machine, but no one considers cars to be robots. In contrast, clinicians and sometimes neuroscientists often confound electromechanical devices with robots [20].

The word “robot” first appeared in 1921 in a science fiction play titled R.U.R. (Rossum’s Universal Robots) written by the Czech author Karel Capek. It derives from the Czech word “robota”, meaning hard workers [19, 26]. The robots invented by Capek were not robots in the popularly understood sense of mechanical devices; instead, they were assembled biological organisms. However, the term has since come to signify primarily electromechanical devices (often humanoid) endowed with artificial intelligence and able to perform a variety of functions, partly through programming and partly through their own ability to act autonomously [27]. According to that, the Robot Institute of America defined a robot as “a programmable, multi-functional manipulator designed to move material, parts or specialized devices through variable programmed motions for the performance of a variety of tasks” [28].

Neurorobotics refers to the branch of science combining neuroscience, robotics, and artificial intelligence. It hence refers to all robots developed for interacting with or for emulating the nervous system of humans or other animals. A neurorobot can be developed for clinical purposes, for example neurorehabilitation or neurosurgery, or for studying the nervous system by emulating its properties, as it occurs for example in the walking robots based on central pattern generators [29].

As mentioned above, a robot should be capable of performing a variety of tasks. This adaptability is based on its on-board sensors, the signals of which are processed by artificial intelligence to change the behaviour of the robot. Hence, the fundamental point differentiating robots from electromechanical devices is the adaptability of their operation. In neurorehabilitation, this differentiation has often been considered as picky, and robots and electromechanical devices are often grouped together during analyses of their efficacy [19]. Treadmills with body weight support and other devices such as Gait Trainer (Reha-Stim) should be defined as electromechanical devices, because, once the physiotherapist has fixed their parameters, they are not capable of autonomously adapting them during operation. Conversely, other devices developed for walking recovery, such as Lokomat (Hocoma, Volketswil, Switzerland), can be defined as robots since they use sensors to adapt their functioning to the patient’s performance (e.g., Lokomat has a position control mode for applying an assistance-as-needed guidance force to the lower limbs).

### Features of Neurorehabilitation Robots

Many neurorehabilitation approaches and techniques have been developed to restore neuromotor function, aiming at the recovery of physiological movement patterns in patients with neurological pathologies. However, none has emerged as a gold standard, since it is common opinion that methods should be specifically tailored for pathologies and patients [30]. However, a common feature of these neurorehabilitative approaches is the need for intensive, repetitive, and task-oriented treatments [25].

Many authors reported that robots can improve rehabilitation outcome. In 2008, Wolbrecht et al. [31] identified three main desirable features for a controller of robot-aided movement training (see Table 1). One year later, Morasso et al. [20] re-stated these features, adding the importance of haptic properties and auto-adaptive capacities. Then, Belda-Lois et al. [30] suggested four features for favoring a top-down approach when a robot is used for post-stroke gait recovery. Finally, Dietz et al. [32] reported four main potential advantages of the use of robots in neurorehabilitation. All these features are listed in Table 1.

**Table 1:** Ideal features of neurorobot

Wolbrecht et al. [29]	Morasso et al. [18]	Belda-Lois et al. [28]	Dietz et al. [30]
High mechanical compliance	High mechanical compliance	Repeatability	Standardized training sessions
Ability to assist patients in completing desired movements	Large range of force	Increased training motivation through use of interactive (bio)feedback	Intensive training
Minimum assistance level	Minimum assistance level	Precisely controllable assistance or resistance during movements	Relieves therapist from physically demanding work
	Soft haptic interaction for proprioceptive awareness	Objective and quantifiable measures of subject performance	Objective and quantifiable measures of subject performance
	Adaptive assistance properties		

The features indicated by Wolbrecht et al. [31] mainly focused on the need of adaptability of neurorobots to patients’ abilities. Morasso et al. [20] added that a robot must have also haptic properties and some intelligent capabilities related to an adaptive assist-as-needed approach. Both studies highlighted the importance of a high mechanical compliance, i.e., the need of having a robot with low-stiffness control. A stiff position controller, such as that of industrial robots, can move limbs along the desired trajectories, limiting errors. However, such a controller impedes error-based learning, which is an essential component of motor re-learning [20]. Furthermore, a low-stiffness robot is potentially less dangerous than a high-stiffness robot during interaction with the patient [20]. Two other studies [30, 32] focused on the importance of intensive (for patients, not therapists) and repeatable exercises. Both pointed out the possibility of exploiting robot sensors not only to adapt to the patient’s performance, but also to provide biofeedback to the patient (increasing his/her motivation and hence participation in rehabilitation), and feedback to therapists and clinicians on patient progress.

Neurorobots have the potential for accurate assessment of motor function in order to assess the patient status, to measure therapy progress, or to give the patient and therapist real-time feedback on movement performance [33]. This approach has been proposed in some recent studies. Kinematic robotic measures, especially those related to range of motion, have recently been indicated as useful in the assessment of motor deficits in reaching movements [34] and proprioceptive function of hands [35] and upper [36] and lower [37] limbs. Furthermore, kinetic robotic measures have been reported as useful in the assessment of upper limb strength [33].

It should be noted as among these features, effectiveness is not listed, probably because it is taken for granted when training is performed in a patient-tailored, intensive, repetitive, and task-oriented manner; however, this issue deserves further attention.

### Effectiveness Paradox in Neurorobotics

Morasso et al. noted a paradox in the assessment of effectiveness of neurorehabilitation robots [20]. Most studies have suggested that robotic treatment should be highly personalized by setting the robot parameters in order to exploit the residual capabilities of each patient for recovering a functional status. This implies that in order to be effective, robotic treatment cannot be standardized, and therefore controlled clinical trials in the traditional sense are impossible, unless aimed at very specific and narrow groups (implying a small sample size, hence poor statistical evidence). The contrast between a standardized treatment (with clear guidelines) allowing the design of a randomized controlled trial (and of clear rehabilitative programmes) with an adaptable treatment, tailored for patients’ capabilities, is the core of this effectiveness paradox. Furthermore, the contrast between standardization and adaptability is not the only problem in designing a methodologically rigorous study. Intensive training may increase the risk of inducing or augmenting spasticity. In addition, the monotony of the same exercise with identical trajectories clashes with the need for continuous adaptation of robots to the changing abilities of patients and with the need for motivating, rather than boring, exercises. Finally, most robots help patients in reproducing a movement that replicates the physiological one, despite the fact that most severely affected patients have a low possibility of a complete recovery.

It should be noted that these inconsistencies are present also in conventional neurorehabilitation training. The scientific bases of neuromotor physiology, neurorehabilitation, and brain plasticity are still not completely clear. Neurorehabilitation is still mainly ill-defined, with competing schools of thought about the best treatment.

This generates another scientific roadblock for neurorobots. In fact, neither the optimal movement tasks nor the optimal mechanical inputs are well known. Therefore, the first problem that a robotics engineer encounters when setting out to build a robotic therapy device is that there is still substantial uncertainty as to what exactly the device should do [38], despite the above-cited general features suggested in the literature.

Interestingly, the scepticism related to neurorobotics due to the rather inconclusive evaluation of its efficacy and to the reported inconsistencies is not mitigated by the consideration that quite similar evaluations could be formulated for the variety of human-delivered rehabilitation techniques [20]. Thus, the doubts about the use of neurorobots could be not only attributed to the uncertainty related to efficacy, but also to some other barriers limiting their wider adoption in rehabilitative settings.

### Other Barriers Limiting Neurorobotics

Other aspects limiting neurorobotics are due to technological, behavioural, and economic barriers [39]. Initial economic burden is a potential limit for robot adoption in neurorehabilitation, although it has been reported that the long-term use of neurorobots can decrease healthcare system costs [20]. For example, a single physiotherapist could manage up to four robots (hence four patients) at the same time [25]. Masiero et al. [40] quantified the cost of using NeReBot (a robot for the treatment of post-stroke upper limb impairment) to be 37 % of the hourly physiotherapy cost, with benefits that include a reduction in hospitalization time. This suggests that robotic technology can be a valuable, and an economically sustainable aid, in the management of patient rehabilitation. Hesse et al. found a similar percentage (41 %) under the assumption that the therapist is needed only at the beginning and end of therapy, and in particular situations where help is needed [41]. In general, rigorous studies on the economic sustainability of robots for neurorehabilitation are very sporadic [42]. These few studies suggest that robotic therapy leads to a reduction of costs for the healthcare system, in terms of a reduction in the hospitalization for each patient, higher autonomy at discharge, or both. However, as highlighted by Turchetti et al. [42], an individual hospital could be less interested than the final payer (e.g., the national or local healthcare system, the private patient, or the insurance companies) in these aspects. However, this clearly depends on the reimbursement regimen and on the agreement between the parties. In general, uncertainty remains about the cost-effectiveness of robotic neurorehabilitation [43].

Technological and behavioural aspects could be related to the possibility that the expectations of patients and clinicians about outcomes of a neurorobotic treatment are too high with regards to the current biomedical engineering level. These reasons seem conceivable, but raise another question: why have such expectations not limited other kinds of medical robot, such as surgical robots? In fact, although surgical robots were introduced at around the same time as neurorehabilitation robots, their benefit in assisting surgery (and especially minimally invasive surgery) is established. Even in fields with no unequivocal evidence of the superiority of robot-assisted over traditional surgery, the popularity and diffusion of robotic surgery has progressively increased [44]. In the last 25 years, robots have brought a tremendous improvement to the field of surgery [45]. Thus, other reasons should be investigated to deeply understand what is still lacking for neurorehabilitation robots in order to match the expectations of patients and clinicians. In this scenario, an irrational aspect seems to play a fundamental role.

### Fear of Robots

In the play of Capek, robots are initially obedient, and, when commanded, they perform the required task, by exactly following human instructions. The robots eventually escape human control and start a rebellion. This theme is similar to the Jewish myth of the Golem of Prague (an animated anthropomorphic being entirely created from inanimate matter) and is used in many science fiction works. Could fear actually play a role in the scepticism towards neurorobots?

In general, studies that used questionnaires to collect the opinions of users (patients and therapists) of neurorehabilitation robots reported good usability, comfort, acceptability, and satisfaction. However, most were feasibility studies that enrolled healthy subjects [46], fewer than 10 patients [47–51], or lacked a control group undergoing conventional physiotherapy [52, 53]. Even when a control group was used, only the satisfaction of experimental physiotherapy was assessed [54]. Hence, these positive results should be read with caution, since they were obtained on a small group of users, often not randomly assigned to robotic therapy. Furthermore, these results can generate a bias, since the patients, who accepted to undergo robotic therapy, could be more trustful with regards to the use of new technological rehabilitation interventions.

In 2000, Burgar et al. reported their experience in developing robots for neurorehabilitation, concluding their work with “we do not view robots as replacements for therapists” [55]. However, most of the initial studies on robots claimed that robotic devices can reduce the number of therapists and the associated costs needed for rehabilitation [25, 56, 57] (despite the existence of cases in which two physiotherapists are required for preparing the most severely affected patients for robotic neurorehabilitation, which is typically the case when harnessing the patient on robots for walking recovery based on body weight support [24]).

Furthermore, in terms of control, the patient’s feelings related to robot use in neurorehabilitation should also be considered. Bragoni et al. [23] identified the level of anxiety of patients as a negative prognostic factor for robotic therapy but not for conventional therapy. In contrast, patients who saw themselves as the chief causal factor in managing their recovery showed higher probability of a better outcome with robotic rehabilitation [23]. This kind of fear could be due to the sensation that robots are not considered trustworthy because they lack human feelings, expertise, and common sense [57]. This is one of the hardest problems in artificial intelligence and robotics faced by bioengineers.

## Three Laws of Neurorobotics

### Three Laws of Robotics

After the play of Capek, robots became iconic, especially thanks to Isaac Asimov’s stories, and to his compilation “I, Robot” in 1950 [58]. In a story included in that compilation and first published in 1942 titled “Runaround”, Asimov invented the three laws of robotics, quoted as being from the “Handbook of Robotics, 56th Edition, 2058”. These rules are a set of fundamental requirements for the design and manufacture of intelligent robots. They are intended to ensure that robots will operate for the benefit of humanity, rather than becoming a threat to humans. These laws had a very influential role in subsequent science fiction works, and became also important with the emergence of robotics as a scientific discipline [59]. The three laws of robotics are:

1. A robot may not injure a human being or, through inaction, allow a human being to come to harm.
2. A robot must obey the orders given it by human beings, except where such orders would conflict with the First Law.
3. A robot must protect its own existence, as long as such protection does not conflict with the First or Second Laws.

These laws define a kind of set of ethic rules for robots (or for the human programmers of their artificial intelligence). The hierarchical structure of these laws places at the first level human health, followed by human will, and finally robot self-preservation. These laws should not be considered only as part of science fiction imagery. Their potential role is so important that they have been re-analyzed in the current context, in the Editorial of a Special Issue of Science, entitled “Robot Ethics” [60]. In this editorial, Sawyer stated that, since the U.S. military is a major source of funding for robotic research, it is unlikely that such laws will be integrated in their design. This argument can be generalized to cover other robotic industries: the development of artificial intelligence is a business, and businesses are usually uninterested in ethical issues. The risk, in the neurorehabilitation field, is that companies may produce attractive robots without proving their effectiveness. The potential risks related to the use of medical robotics deserve attention: harm may occur from anomalous functioning, or even from normal robot behaviour [57]. If many of the problems related to neurorobots are related to fear, risks, and ethical issues, it is probably time to define a set of rules for neurorobot ethics before defining their desirable features.

### Three laws of neurorobotics

According to the aforementioned desirable features of a neurorobot, we have re-formulated the three laws of robotics into three laws for robotics in neurorehabilitation:

1. A robot for neurorehabilitation may not injure a patient or allow a patient to come to harm.
2. A robot must obey the orders given it by therapists, except where such orders would conflict with the First Law.
3. A robot must adapt its behavior to patients’ abilities in a transparent manner as long as this does not conflict with the First or Second Law.

These laws and their implications are discussed below.

## Discussion

### First Law of Neurorobotics: Need for High Benefit/risk Ratio

Personal care robots (e.g., mobile servant robots, physical assistant robots, and person carrier robots) should be designed in accordance with the international standards defined by ISO 13482:2014 [61]. In 2014, the International Organization for Standardization published these criteria for designing personal care robots, providing the needed requirements to eliminate or reduce the risks associated with the use of medial robots to an acceptable level. ISO 13482:2014 is more specific for personal care robots, including neurorobots, than the previous ISO14971:2000 [62]. ISO 13482:2014 can be considered to be in line with the first law of Asimov, with “harm” referring to that to the patient. Datteri [52], in a review about responsibility in using medical robots (including surgery and diagnostic robots, neurorehabilitation robots, robotic prostheses, and even next-generation personal assistance robots), stated that these devices operate in close proximity or direct physical contact with humans, manipulate instruments inside the patient’s body or directly move user’s impaired limbs, and have invasive or non-invasive connections with the human nervous system. They can hence contribute to improving the precision of medical treatments, relieving therapists of tasks that require considerable accuracy and physical effort, and improving the quality of life of patients [63]. Nevertheless, they also may threaten the physical integrity of patients, not only through harmful events caused by anomalous behaviours (e.g., in surgery), but even through normal operation [57]. This can typically occur for neurorehabilitation robots whose efficacy has not been proven [57]. Datteri’s review gives the example of Lokomat, showing that, despite its diffusion in many rehabilitation centers, there is neither well-supported experimental nor theoretical evidence that Lokomat-based therapies are at least as beneficial as conventional therapies. Instead, the review gives examples of studies that showed that Lokomat reproduces abnormal and non-physiological gait patterns due to the restriction of pelvis movement, altering lower limb joint kinematics [64] and muscle activations [65]. This limitation has recently been overcome in Lokomat^®^Pro (Hocoma) by the addition of an optional module that allows lateral translation and transverse rotation of the pelvis, aiming at a more physiological movement. However, it is still unclear if training based on physiological movement is the optimal solution for patients severely affected and probably unable to completely recover physiological patterns. In fact, recovery of autonomy in walking should be the objective of robotic gait rehabilitation, where recovery of physiological gait patterns is not mandatory.

Neurorobots should be safe not only in terms of movement, but also from other medical points of view. For example, despite the variety of gait patterns, robotic gait training performed with body weight support has only recently been proven safe for training intensive walking in non-autonomous ambulatory patients with subacute stroke. The reason is that the cardio-respiratory demand is lower than that in conventional walk training performed overground [66]. Interestingly, the authors found the opposite result for healthy subjects: overground walking was less demanding than robotic walking. They suggested that this could have been because the robot imposes non-natural trajectories, which force subjects to activate non-natural sensorimotor walking patterns.

We would like to enlarge the meaning of “harm” to all possible damage to patients. Time spent on an ineffective, slightly effective, or even detrimental robot should be considered as damage, because the patient could spend the same time in a more effective treatment. Hence, the first law implies that robot usage should be at least as safe and effective as other treatments, meaning that it should have a higher benefit-risk ratio than that of human-administered treatments. This ratio should be evaluated before commercialization of the device, and not afterwards, as is often done currently.

But how can a robot be effective in the light of the cited effectiveness paradox and in the absence of a clear scientific background? Firstly, it is probably time to delay the commercial launch of neurorobots until a deep examination of their potential effectiveness is conducted, adopting an approach more similar to that used in other medical or engineering disciplines. For example, specific rules are defined for clinical trials prior to drug commercialization (Table 2). These trials require Phase I, (commonly performed in the producer laboratories), followed by Phases II and III (performed in independent hospitals), before commercialization can occur. Further, Phase IV follows in clinical or daily living settings. Dobkin redefined these phases for motor rehabilitation treatments [67] (refer to Table 2), and we suggest that a similar roadmap should be followed by companies before commercialization of neurorobots (that should occur only after an equivalent Phase III).

**Table 2:** Clinical trial phases in drug commercialization and motor rehabilitation

Phase	Drug commercialization	Studies on rehabilitation	Purpose
Phase I	*Checking for safety* (on 10–20 healthy volunteers)	*Consideration-of-concept studies* (on 6–12 patients)	To test concepts and related safety on animals or on a small group of patients
Phase II	*Checking for efficacy* (on about 200 patients)	*Development of Concept Trials* (>15 patients)	To standardize the new intervention and add a control group, randomization, and masked outcomes. To establish the best dose of therapy. To assess sample size
Phase III	*Confirmation of findings in large patient population* (>1000 patients for detecting rare side effects)	*Demonstration of Concept Trials* (on a sample with a properly computed size)	To prove effectiveness and safety of intervention
Phase IV	*Testing long-term safety* (real life patients)	*Proof of concept* (multicenter randomized clinical trials)	To establish generalizable efficacy and safety

Furthermore, for neurorehabilitation robots, there is still a lack of clear information about how to administer robotic therapy, proper use, treatment duration and frequency, precautions, possible side effects, etc. However, the effectiveness of a treatment (including that with a neurorehabilitation robot) depends on the patient characteristics (e.g., type and severity of disease, presence of specific deficits) [16], on the duration and frequency of sessions to administer, and on the correct phase of rehabilitation at which the therapy should be administered [25]. For example, Morone et al. reported that patients with more severe impairments in the motor leg benefited more from robotic-assisted therapy than did patients with greater voluntary motor function in the affected limb, who can perform intensive and less constrained training in conventional therapy [21, 22]. Unfortunately, neurorobot handbooks are at the moment still similar to generic commercial pamphlets, far from drug information sheets.

### Second Law of Neurorobotics: Tool for Therapists

Some therapists see a robot as a possible substitute for their work. Morasso et al. thus titled their review on robots for rehabilitation “Desirable features of a ‘humanoid’ robot-therapist” [20]. Hidler et al. emphasized that the goal of introducing robots into rehabilitation hospitals is not to replace therapists, but rather to complement existing treatment options [56]. Nevertheless, it is reasonable to believe that the reduction of healthcare costs is at least one of the main motives driving research in neurorobotics [57], given that many studies have reported that robots may reduce the cost of rehabilitation by reducing the number of required therapists [25, 56, 57].

The higher popularity of neurosurgery robots compared to neurorehabilitation robots is thus likely due to the fact that the former do not replace the surgeon, but aid him. Similarly, a robot for rehabilitation should not be considered as a standing-alone rehabilitation device [68], but a tool in the hands of therapists, giving them more precise movements, more intensive, repeatable, or adaptable patterns, according to the therapists’ expertise, and relieving them from fatigue. The therapist should therefore be included in the loop, in order to drive the symbiotic equilibrium between robot and patient towards an optimum, by dialoguing with the patient, motivating them, and getting verbal feedback on fatigue, pain, and emotional stress (parameters difficult to monitor with sensors) [57]. Recently, the need for a therapist as motivator to avoid the patient having a passive role during robotic therapy has been overcome by a top-down approach of robots combined with stimulating biofeedback, video-game-based therapy, and even brain-computer interfaces [19, 30]. However, a therapist should play a key role in terms of robotic therapy administration, such as robot parameter adjustments, avoiding harmful patient compensation strategies, identification of the trade-off between challenging tasks that help rehabilitation and those that demoralize patients.

**Fig. 1:**
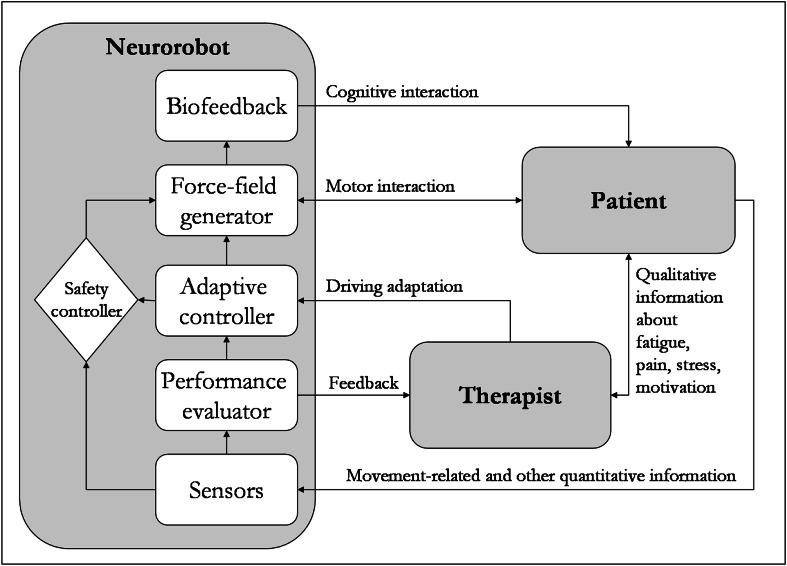
Ideal patient-therapist-robot loop

To this end, we propose to extend the loop proposed by Morasso et al. [20] to include the therapist (see Fig. 1). In our opinion, the desired reduction of costs for the healthcare system can be obtained not by reducing the number of therapists, but increasing the efficacy of rehabilitation, reducing the length of stay in rehabilitative hospitals, and releasing more autonomous patients with a consequent reduction of home care costs.

The proposed second law of neurorobotics, making the robot perfectly obedient to the therapists’ requests, may seem obvious, but it is not. Besides the above-mentioned problems related to non-physiological gait patterns in Lokomat-based therapy [57], another example of robot “disobedience” is the discrepancy between the desired and actual values of some parameters of the electromechanical Gait Trainer (as highlighted in [24]). The effective percentage of body weight supported by the machine is different from that selected in the initial static condition, since the machine does not take into account the changes that occur in the patient capacity to support their own weight during training. Furthermore, the authors highlighted that for Gait Trainer, the defined selector of walking speed is actually a selector of step duration, and that the reported speed coincides with the real one only if the maximum step length has been also selected.

Robots should “disobey” clinicians’ orders only if their sensors indicate that such orders lead to a potential risk for the patient. This highlights the importance of sensors, which is at the base of the adaptability and autonomy of any robotic system [28]. In contrast, an electromechanical device is not required to detect a potentially dangerous choice by therapists due to wrong parameter tuning.

### Third Law of Neurorobotics: Artificial Intelligence as Support for Human Intelligence

The presence of a therapist in the loop (Fig. 1) allows human control of the device, but the robot’s artificial intelligence should not be limited to the safety control of human decisions. During rehabilitation, there are many parameters to calibrate, tune, and adapt. Firstly, the clinician should always consider the effects of a parameter change on other parameters. For example, to increase speed during overground walking, a subject can reduce step duration, increase step length, or both (usually at the same time). In Lokomat-based training, when a therapist increases the patient’s walking speed, they are actually reducing the step duration without altering the step length, since this parameter depends on the sagittal range of hip motion; such changes in that hip range of motion need a manual adjustment by the therapist. The handbook of Hocoma [69] suggests that therapists should consider the following points when increasing speed: (1) manually adapt step length acting on hip range of motion controller (the wider is the hip movement, the longer is the step); (2) adjust the synchronization between treadmill and exoskeleton speed (automatic setting is also possible); (3) adjust the hip offset (not only range); (4) take into account that foot impact could increase, and hence increase the load on the joints; (5) check the quality of the movement that may be affected by the change. This highlights how many parameters are related to a simple change of speed in a robot for gait training. Furthermore, speed is a parameter with a very clear physiological meaning. More problems could occur for a parameter for which it is not so easy to understand its role, such as guidance force.

Robot artificial intelligence should be capable of automatically performing all the control changes required by the therapist, while providing them with a clear quantitative overview of all these changes. The adoption of robotic technologies for helping patients and therapists and quantitatively evaluating patient recovery is the main issue of European projects such as MAAT (“Multimodal interfaces to improve therapeutic outcomes in robot-assisted rehabilitation”, www.echord.info/wikis/website/maat) and SYMBITRON (“Symbiotic man–machine interactions in wearable exoskeletons to enhance mobility for paraplegics”, www.symbitron.eu). These projects include the patient in a symbiotic loop with the robot, similarly to what we suggest in Fig. 1. Then, the therapist should simply be required to qualitatively control patient performance under the new conditions.

Summarising these concepts: a new generation of human–machine interfaces integrated in neurorobots should be developed, in which the therapist’s commands at the macro level can be translated in micro changes autonomously by the robot, which should inform the therapist of these changes. However, there are no easy ways to assess algorithmically whether the mutual patient-robot adaptation is the optimal one for favouring the neuromotor recovery [57]. For this reason, the therapist should be kept in the loop. In contrast with the robot, the therapist has a qualitative but natural access to the health status of the patient. For instance, they have detailed feedback of feelings and sensations by dialoguing with the patient.

## Conclusion

Most studies and reviews about robots for neurorehabilitation have focused on their effectiveness, but have found inconsistent results. Little attention has been given to robot ethics, probably because artificial intelligence is still primitive. However, data shows that patients and therapists are somewhat afraid of robots. Although we did not suggest new technical solutions, in this review, we described the state of the art of robots for neurorehabilitation, and suggested a set of rules, which are a re-formulation of Asimov’s three laws of robotics. We indicated the need for these laws with many examples. The proposed three laws of neurorobotics highlight the ethical need to prove a robot’s effectiveness before commercialization, as well as the desirable features that neurorobots should have. Furthermore, we highlighted the need for including the therapist in the loop between patient and robot. Finally, we suggested that neurorobots can be a valuable tool in therapists’ hands, helping them not only in repetitive and intensive patient mobilization, but also providing quantitative information about a patient’s deficits, residual abilities, and functional recovery. We think that these three laws should be considered from the first stages of neurorobot design. They may bring together engineers and clinicians for the development of a new, effective generation of robots for neurorehabilitation.
